# Extending the data collection from a clinical trial: The Extended Salford Lung Study research cohort

**DOI:** 10.1038/s41533-022-00322-7

**Published:** 2023-01-18

**Authors:** Wilhelmine Meeraus, Qinggong Fu, George Mu, Mark Fry, Lucy Frith, Jeanne M. Pimenta

**Affiliations:** 1 GSK, Brentford, United Kingdom; 2 GSK, Collegeville, PA USA

**Keywords:** Asthma, Chronic obstructive pulmonary disease

## Abstract

The Extended Salford Lung Study (Ext-SLS) is an extension of the Salford Lung Studies (SLS) in asthma and chronic obstructive pulmonary disease (COPD) through retrospective and prospective collection of patient-level electronic health record (EHR) data. We compared the Ext-SLS cohort with the SLS intention-to-treat populations using descriptive analyses to determine if the strengths (e.g. randomization) of the clinical trial were maintained in the new cohort. Historical and patient-reported outcome data were captured from asthma-/COPD-specific questionnaires (e.g., Asthma Control Test [ACT]/COPD Assessment Test [CAT]). The Ext-SLS included 1147 participants (*n* = 798, SLS asthma; *n* = 349, SLS COPD). Of participants answering the ACT, 39% scored <20, suggesting poorly controlled asthma. For COPD, 61% of participants answering the CAT scored ≥21, demonstrating a high disease burden. Demographic/clinical characteristics of the cohorts were similar at SLS baseline. EHR data provided a long-term view of participants’ disease, and questionnaires provided information not typically captured. The Ext-SLS cohort is a valuable resource for respiratory research, and ongoing prospective data collection will add further value and ensure the Ext-SLS is an important source of patient-level information on obstructive airways disease.

## Purpose

Obstructive airway diseases are a leading cause of morbidity and mortality worldwide^1,2^. Chronic obstructive pulmonary disease (COPD) is the most prevalent chronic respiratory disease globally, followed by asthma, and both are associated with significant illness, disability, and mortality^3,4^. Understanding the real-world patient experience of these conditions is crucial to providing appropriate care and treatment^5^, particularly when taking a “treatable traits” approach to disease management^6^. However, there are few longitudinal research cohorts available with well-characterized, real-world data. Here, we describe a new research cohort, the Extended Salford Lung Study (Ext-SLS), which combines clinical trial data from asthma and COPD patients with electronic health record (EHR) data and detailed, self-reported, information to assess the impact of respiratory disease on patients’ everyday lives. The aim of the Ext-SLS was to gain a more in-depth and long-term understanding of the patients’ disease journey and improve scientific and clinical understanding of COPD/asthma disease risk, treatment, and progression.

## Methods

### Ethics statement

Ethical approval was granted by the North West—Greater Manchester East Research Ethics Committee (REC number: 17/NW/0122). Patients enrolled in the Ext-SLS provided written informed consent for the collection of data via their EHRs both retrospectively to the earliest available record and prospectively for ≤10 years from consent into the study.

### The Ext-SLS

The Ext-SLS is an extension study of the SLS. The SLS were first-of-their-kind, Phase IIIb, pragmatic, randomized, controlled clinical trials designed to evaluate the safety and effectiveness of initiating fluticasone furoate/vilanterol (FF/VI; 100–200/25 µg) versus continuing usual care (UC)^7–9^. Patients were recruited from general practice clinics in Salford and Greater Manchester, UK, between March 2012 and October 2014 for SLS COPD, and November 2012 and December 2016 for SLS asthma. Patients consented to relevant data being collected from up to 3 years prior to the study and for the 12-month interventional treatment period. Participants were broadly representative of COPD and asthma populations in the United Kingdom (UK)^7–9^.

Participants were eligible for inclusion in the Ext-SLS if they were randomized to treatment in the original SLS and were able to provide written consent for data collection in the Ext-SLS. Patient recruitment (conducted 2018–2019) was general practitioner (GP) led. Participants consented to share primary care EHR data held by GP sites, as well as secondary care EHR data obtained from the NHS Digital Hospital Episodes Statistics databases covering admitted patient care, outpatient visits and Accident and Emergency attendances^10^.

Primary care EHRs in the Ext-SLS include all non-sensitive and coded information relating to patient demographics, prescriptions, symptoms, diagnoses, referrals to and interactions with secondary care, preventative healthcare, diagnostic testing, and lifestyle information (e.g., smoking, alcohol use). Primary care EHR data are coded using Read, Systematized Nomenclature of Medicine (SNOMED) and local Egton Medical Information Systems (EMIS) codes^11^.

Retrospective EHR data were collected to the earliest available record and prospective data are being collected periodically for up to 10 years from the date of consent.

Participants completed disease-specific questionnaire booklets that captured information about their disease histories and management at the point of Ext-SLS consent. The questionnaires gathered information not available in EHRs and included validated disease-specific instruments in addition to questions on early life experience, environmental exposures, medication use, physical functioning, and social support (Figs. S1, S2). Validated instruments are described in Table 1.

**Table 1:** Validated instruments included in the disease-specific questionnaires.

Test	Condition assessed	Description	Validation reference
**COPD and Asthma Sleep Impact Scale (CASIS)**	Asthma and COPD	A seven-question instrument, with five items relating to disturbance falling asleep or staying awake during the day. The remaining items concern sleep quality. Responses range from 1 = never to 5 = very often, with two items reverse-scored. The sum of raw scores is linearly transformed to arrive at a total score ranging from 0 to 100, with higher scores indicating worse sleep quality. The CASIS has a look-back period of 1 week.	(Pokrzywinski et al., 2009)^22^
**Asthma Control Test (ACT)**	Asthma	A five-item assessment of control based on asthma symptom severity/frequency and rescue medication use. Each question is assigned a value of 1 to 5. Total scores of >19 indicate controlled asthma, with scores of <16 indicating uncontrolled asthma (scores of 16–19 represent somewhat controlled asthma and scores of <16 represent poorly controlled asthma). The ACT has a look-back period of four weeks.	(Schatz et al., 2007)^23^
**Asthma Control Questionnaire-6 (ACQ-6)**	Asthma	A six-item assessment of control based on asthma symptom severity/frequency and rescue medication use. For each question, a score of 0 indicates no impairment and 6 indicates maximum impairment. The total score is the mean of responses to all six questions. A total score of 0.0–0.75 is classified as well-controlled asthma; 0.75–1.5 as a “gray zone”; and >1.5 as poorly controlled asthma. The ACQ has a look-back period of 1 week.	(Juniper et al., 2005)^26^
**COPD Assessment Test (CAT)**	COPD	An eight-item questionnaire assessing the severity and impact of COPD symptoms. Each question is scored from 0–5, with total scores ranging from 0 to 40. Scores >20 indicate a high impact of COPD on a patient’s life and scores <10 indicate a low impact.	(Jones et al., 2009)^21^
**Modified Medical Research Council dyspnea scale (mMRC)**	COPD	A five-point scale (0 [low impact]–4 [high impact]) measuring the degree and impact of breathlessness.	(Hajiro et al., 1998)^20^

*COPD* chronic obstructive pulmonary disease.

### Data analysis

To understand whether the Ext-SLS cohort was representative of the wider SLS population, we report descriptive comparisons of the Ext-SLS and SLS intention-to-treat (ITT) cohorts using data captured at entry to the original SLS. Post-hoc analysis was performed using chi-square testing to examine differences in select baseline characteristics. The ITT populations of the SLS included patients who were randomized in the studies and received at least one prescription of study medication (FF/VI or UC). The generalizability of the COPD population to the non-trial COPD population has been assessed previously^12^.

A descriptive analysis of the Ext-SLS primary care EHR and questionnaire data was conducted using *N* and % for categorical data; mean and standard deviation (SD) or median and inter-quartile range for continuous data. Secondary care data were not available at the time of analysis. Description of primary care data focused on current lifestyle information (smoking, body mass index [BMI]), comorbidities, and blood tests (eosinophils, neutrophils) recorded within 24 months prior to the Ext-SLS consent date. For participants with COPD, modified Medical Research Council (mMRC) dyspnea score, Global Initiative for Chronic Obstructive Lung Disease (GOLD) grade of airflow limitation (1–4) and lung function (forced expiratory volume in one second [FEV_1_]) were assessed. Questionnaires were analyzed on a per-question basis using all “known” answers (ie, where an answer other than “Don’t know” was provided) for each specific question. Additionally, questionnaire data were analyzed using the subset of participants that completed all required questions (i.e., not part of a skip pattern).

### Reporting summary

Further information on research design is available in the Nature Research Reporting Summary linked to this article.

## Results

### The Ext-SLS study population

Overall, 1183/7032 participants (18.9%) from the SLS ITT population consented to the Ext-SLS and 1147 of these (*n* = 798 asthma, *n* = 349 COPD) completed questionnaires and had primary care data available for analysis. The SLS ITT populations included 4233 asthma patients and 2799 COPD patients. While the intention was to collect full retrospective EHR data, ultimately, only ~10 years of data (including retrospective and some prospective) data were obtained and are available. Participants consented to the Ext-SLS a mean of 3.2 years post-SLS. In the SLS, patients were randomized 1:1 to FF/VI versus UC. In the Ext-SLS, 46% (368) of participants with asthma and 48% (167) of participants with COPD were originally randomized to FF/VI during the SLS. Most Ext-SLS participants were from Salford (51%; *n* = 582) or Trafford (30%; *n* = 341) and provided consent in 2018 (91%; *n* = 1039).

### Descriptive comparison of Ext-SLS and SLS ITT populations

Baseline demographics collected at the beginning of the SLS were broadly similar between the Ext-SLS and SLS cohorts (Table 2), with some exceptions. Among participants with asthma, the proportion aged >50 years at baseline was higher in the Ext-SLS cohort compared with the SLS cohort (*p* < 0.0001). Conversely, at entry to the SLS, participants in the Ext-SLS cohort with COPD were, on average, slightly younger at baseline than in the SLS COPD ITT population, with a greater proportion of participants aged <50 years (*p* = 0.0013). Compared with SLS asthma participants, there were more females in the Ext-SLS (*p* = 0.0035), and a larger proportion reported never smoking at baseline (*p* = 0.007). For participants with COPD, there was a greater proportion of “ex-smokers” and a smaller proportion of “current smokers” in the Ext-SLS cohort compared with the SLS cohort; however, these differences were not statistically significant.

**Table 2:** Baseline characteristics of the Ext-SLS cohort and SLS ITT populations, as recorded at the time of entry into the SLS.

	Asthma		COPD	
	Ext-SLS population (*N* = 798)	SLS ITT population (*N* = 4233)	Ext-SLS population (*N* = 349)	SLS ITT population (*N* = 2799)
**Sex,** ***n*** **(%)**				
Female	515 (64.5)	2498 (59.0)	173 (49.6)	1369 (48.9)
Male	283 (35.5)	1735 (41.0)	176 (50.4)	1430 (51.1)
**Age, year**s				
Mean (SD)	53.4 (14.8)	49.8 (16.4)	65.4 (8.8)	66.7 (9.9)
Range	18–88	18–91	41–91	40–93
**Age group,** ***n*** **(%)**				
18 to 50 years	318 (39.8)	2151 (50.8)	242 (69.3)	1693 (60.5)
>50 years	480 (60.2)	2082 (49.2)	107 (30.7)	1106 (39.5)
**Body Mass Index (BMI), kg/m**^**2**^				
N with BMI available	790	4152	311	2231
Mean (SD)	29.9 (6.9)	29.9 (6.9)	28.1 (6.2)	27.8 (6.5)
Range	17–70	15–70	16–50	12–77
**Smoking status,** ***n*** **(%)**				
Current smoker	109 (13.7)	849 (20.1)	146 (41.8)	1289 (46.1)
Ex-smoker	261 (32.7)	1366 (32.3)	184 (52.7)	1375 (49.1)
Never smoker	427 (53.5)	1988 (47.0)	19 (5.4)	135 (4.8)
**Medical conditions at baseline**				
Any condition, *n* (%)	335 (42.0)	1625 (38.4)	262 (75.1)	2145 (76.6)
Cardiovascular risk factors, *n* (%)	214 (26.8)	1094 (25.8)	188 (53.9)	1448 (51.7)
Hypercholesterolemia	180 (22.6)	917 (21.7)	181 (51.9)	1298 (46.4)
Diabetes mellitus	68 (8.5)	406 (9.6)	36 (10.3)	438 (15.6)
Vascular disorders, *n* (%)	236 (29.6)	1099 (26.0)	170 (48.7)	1363 (48.7)
Hypertension	236 (29.6)	1098 (25.9)	169 (48.4)	1355 (48.4)
Cerebrovascular accident	4 (<1)	18 (<1)	2 (<1)	27 (<1)
Cardiac disorders, *n* (%)	56 (7.0)	346 (8.2)	67 (19.2)	720 (25.7)
Coronary artery disease	27 (3.4)	221 (5.2)	50 (14.3)	555 (19.8)
Arrhythmia	30 (3.8)	144 (3.4)	18 (5.2)	239 (8.5)
Congestive heart failure	5 (<1)	47 (1.1)	13 (3.7)	164 (5.9)
Myocardial infarction	3 (<1)	8 (<1)	NR	7 (<1)
Asthma, *n* (%)	–	–	69 (19.8)	609 (21.8)

*COPD* chronic obstructive pulmonary disease, *Ext-SLS* Extended Salford Lung Study, *ITT* intention to treat, *SD* standard deviation, *SLS* Salford Lung Study.

Respiratory disease characteristics for asthma (Table 3) and COPD (Table 4) were broadly similar between the SLS and Ext-SLS cohorts, but with some notable differences. Participants with asthma in the Ext-SLS potentially had less severe disease at SLS entry, compared with the SLS asthma cohort, based on self-reported symptoms, control as measured using the ACT and short-acting β_2_-agonist (SABA) reliever therapy use (Table 3). Participants with COPD in the Ext-SLS had slightly better lung function, lower CAT scores (a greater proportion of participants with scores below 10), and a smaller proportion of the Ext-SLS cohort were GOLD Grade 3 or 4 (Table 4).

**Table 3:** Asthma disease characteristics in the Ext-SLS cohort and SLS ITT populations, captured at entry to the SLS.

	Ext-SLS Population (*N* = 798)	SLS ITT Population (*N* = 4233)
**Duration of asthma,** ***n*** **(%)**^**a**^		
<6 months	10 (1.3)	54 (1.3)
≥6 months to <1 year	11 (1.4)	77 (1.8)
≥1 to <5 years	82 (10.3)	438 (10.3)
≥5 to <10 years	97 (12.2)	519 (12.3)
≥10 years	598 (74.9)	3144 (74.3)
**Any daytime symptoms more than twice a week?,** ***n*** **(%)**		
Yes	708 (88.7)	3830 (90.5)
No	90 (11.3)	403 (9.5)
**Use of SABA more than twice a week?,** ***n*** **(%)**		
Yes	551 (69.0)	3044 (71.9)
No	247 (31.0)	1189 (28.1)
**Any limitations of activities in the past week?,** ***n*** **(%)**		
Yes	385 (48.2)	2162 (51.1)
No	413 (51.8)	2071 (48.9)
**Any nocturnal symptoms/awakening in past week?,** ***n*** **(%)**		
Yes	393 (49.2)	2117 (50.0)
No	405 (50.8)	2116 (50.0)
**Total number of exacerbations (any severity) in the year prior to randomization,** ***n*** **(%)**		
0	513 (64.3)	2692 (63.6)
1	183 (22.9)	973 (23.0)
≥2	102 (12.8)	568 (13.4)
Mean (SD)	0.6 (1.2)	0.6 (1.1)
Range	0–12	0–14
**Number of severe asthma exacerbations in year prior to randomization,** ***n*** **(%)**		
0	513 (64.3)	2692 (63.6)
≥1	285 (35.7)	1541 (36.4)
**ACT score,** ***n*** **(%)**		
≥20	263 (33.0)	1210 (28.6)
16–19	250 (31.3)	1307 (30.9)
≤15	285 (35.7)	1716 (40.5)
**Asthma symptoms,** ***n*** **(%)**		
<2 questions answered yes	141 (17.7)	634 (15.0)
≥2 questions answered yes	657 (82.3)	3599 (85.0)

*ACT* Asthma Control Test, *Ext-SLS* Extended Salford Lung Study, *ICS* inhaled corticosteroid, *ITT* intention to treat, *SABA* short-acting β_2_-agonist, *SD* standard deviation, *SLS* Salford Lung Study.

^a^*n* = 4232.

**Table 4:** COPD disease characteristics in the Ext-SLS cohort and SLS ITT populations, captured at the entry to the SLS.

	Ext-SLS Population (*N* = 349)	SLS ITT Population (*N* = 2799)
**Duration of COPD,** ***n*** **(%)**		
<5 years	185 (53.0)	1319 (47.1)
≥5 to <10 years	103 (29.5)	975 (34.8)
≥10 to <15 year	31 (8.8)	321 (11.5)
≥15 to <20 years	13 (3.7)	98 (3.5)
≥20 to <25 years	9 (2.6)	49 (1.8)
≥25 years	3 (<1)	37 (1.3)
**COPD type,** ***n*** **(%)**		
Chronic bronchitis	18 (5.2)	137 (4.9)
Emphysema	50 (14.3)	398 (14.2)
Chronic bronchitis and emphysema	7 (2.0)	37 (1.3)
Not diagnosed	274 (78.5)	2226 (79.5)
**Number of moderate and severe exacerbations**^**a**^ **during the year prior to randomization**		
0	68 (19.5)	530 (18.9)
1	130 (37.2)	902 (32.2)
≥2	150 (43.3)	1367 (48.9)
Mean (SD)	1.85 (1.9)	2.01 (2.0)
Range	0–14	0–15
**Number of moderate exacerbations**		
0	74 (21.2)	568 (20.2)
≥1	275 (78.8)	2231 (79.7)
**Number of severe exacerbations**^**a**^*n* (%)		
0	332 (95.1)	2613 (93.4)
≥1	17 (4.9)	186 (6.6)
**Post-bronchodilator FEV**_**1**_ **(L)**^**b**^		
Mean (SD)	1.744 (0.6)	1.619 (0.6)
Range	0.43–3.88	0.33–4.31
**Post-bronchodilator percent predicted FEV**_**1**_ **(%)**^**b**^		
Mean (SD)	63.44 (17.8)	60.68 (19.1)
Range	10.8–111.5	10.8–129.1
**Post-bronchodilator FVC (L)**^**b**^		
Mean (SD)	3.188 (1.0)	2.993 (1.0)
Range	1.14–6.41	0.62–7.06
**Post-bronchodilator FEV**_**1**_**/FVC (%)**^**b**^		
Mean (SD)	54.79 (12.4)	54.52 (13.4)
Range	23.6–82.5	18.3–98.6
**GOLD category at baseline,** ***n*** **(%)**^**b**^		
GOLD Grade 0	31 (11.1)	268 (12.2)
GOLD Grade 1 or 2	188 (67.1)	1293 (58.8)
GOLD Grade 3 or 4	61 (21.8)	638 (29.0)
**CAT score at baseline,** ***n*** **(%)**^**c**^		
<10	50 (14.3)	286 (10.2)
≥10	299 (85.7)	2510 (89.8)

*CAT* COPD Assessment Test, *COPD* chronic obstructive pulmonary disease, *Ext-SLS* extended Salford Lung Study, *FEV*_*1*_ forced expiratory volume in one second, *FVC* forced vital capacity, *GOLD* global initiative for chronic obstructive lung disease, *ITT* intention to treat, *SD* standard deviation, *SLS*, Salford Lung Study.

^a^Severe exacerbations are defined as those requiring treatment with systemic corticosteroids, antibiotics, or leading to hospital attendance.

^b^Ext-SLS *n* = 280, SLS ITT *n* = 2199.

^c^Ext-SLS *n* = 349, SLS ITT *n* = 2796.

### Description of select Ext-SLS primary care data

A selection of variables relevant to asthma and COPD are described here, illustrating the breadth of information available.

Table 5 shows BMI measurements recorded in participants’ primary care EHR; most asthma and COPD participants were overweight (BMI ≥25 kg/m^2^) or obese (BMI ≥30 kg/m^2^). Average BMI measurements in Ext-SLS (at or just prior to the time of consent) reflected measurements recorded at SLS entry (asthma 29.9 and 29.9; COPD 28.1 and 27.8, respectively; Table 2).

**Table 5:** Most recent BMI result and self-reported smoking status recorded in primary care EHR in the 24 months prior to Ext-SLS consent.

	Asthma (*N* = 798)	COPD (*N* = 349)
**BMI,** ***n*** **(%)**		
<18.5	6 (<1)	18 (5.2)
18.5–<25	172 (21.6)	102 (29.2)
25–<30	259 (32.5)	123 (35.2)
≥30	278 (34.8)	85 (24.4)
unknown	83 (10.4)	21 (6.0)
**Smoking status,** ***n*** **(%)**		
Ex	440 (55.1)	199 (57.0)
Never	201 (25.2)	8 (2.3)
Current	108 (13.5)	131 (37.5)
Unknown	49 (6.1)	11 (3.2)
Use of nicotine supplement^a^	4 (<1)	7 (2.1)
Transition from current to ex-smoker from randomization date to current status in Ex-SLS^b^	71 (46)	48 (31)

*BMI* body mass index, *COPD* chronic obstructive pulmonary disease, *EHR* electronic health record, *Ext-SLS* extended Salford Lung Study.

^a^ % Nicotine use based on all current and ex-smokers.

^b^ % transition based on all current smokers at baseline.

The smoking status of participants in the Ext-SLS showed that, among participants who had been smokers at SLS baseline, 46% of those with asthma and 31% of those with COPD had stopped smoking (Table 5).

Common conditions (ever recorded in EHR) among Ext-SLS asthma participants included atopy (57.0%), pneumonia (53.8%), and diabetes mellitus (14.9%) (Table 6). Evidence of current asthma (defined based on the presence of an asthma medical code in the 12 months preceding Ext-SLS consent) was observed in 86.2% of the asthma group. Two participants had no medical information available, meaning 99.7% of the asthma group were confirmed as having ever had asthma (i.e., any record of asthma prior to Ext-SLS consent).

**Table 6:** Select medical conditions ever recorded in Ext-SLS primary care EHR data up to and including the date of Ext-SLS consent.

Medical conditions, *n* (%)	Asthma (*N* = 798)	COPD (*N* = 349)
**Asthma current**^**a**^	688 (86.2)	75 (21.5)
**Asthma ever**	796 (99.7)	219 (62.8)
**Arrhythmia**	111 (13.9)	91 (26.1)
**Atopy**	455 (57.0)	129 (37.0)
**Congestive heart failure**	14 (1.8)	32 (9.2)
**Coronary artery disease**	62 (7.8)	83 (23.8)
**Diabetes mellitus**	119 (14.9)	71 (20.3)
**Myocardial infarction**	20 (2.5)	30 (8.6)
**Nasal polyp**	43 (5.4)	16 (4.6)
**Pneumonia**	429 (53.8)	239 (68.5)
**Sarcopenia**	2 (<1)	–

*COPD* chronic obstructive pulmonary disease, *EHR* electronic health record, *Ext-SLS* extended Salford Lung Study.

^a^Asthma medical code within 12 months prior to Ext-SLS consent date.

Among Ext-SLS COPD participants, comorbidities included pneumonia (68.5%), atopy (37.0%) and arrhythmia (26.1%). In the 12 months prior to Ext-SLS consent, 21.5% of COPD participants had evidence of current asthma, while 37.2% had no prior asthma diagnosis (Table 6).

A large proportion of asthma and COPD participants had records for blood eosinophil (66 and 83%, respectively) and neutrophil counts (68 and 86%, respectively); with many having multiple measures (mean 2.2 asthma, 2.6 COPD) in the 24 months prior to Ex-SLS consent. Compared with asthma participants, a slightly higher proportion of COPD participants had blood eosinophil counts ≥150/μl (69 versus 66%). Neutrophil counts <6000/μl were recorded for 88% of asthma participants and 77% with COPD.

Among the 349 Ext-SLS COPD participants, 75% had information on GOLD airflow grade; most participants were GOLD Grade 2 (43%). An average of three (range 1–12) FEV_1_ measurements were taken in the 24 months prior to consent.

Prescriptions of respiratory medicines in the 12 months prior to Ext-SLS consent are detailed in Table 7. A high proportion of asthma and COPD participants were prescribed SABA (83 and 90%, respectively). This was also the case for inhaled corticosteroid/long-acting ß2-agonist (ICS/LABA) fixed-dose combinations (78 and 88%, respectively). Leukotriene receptor antagonists were prescribed to 15% of asthma participants, while 25% of COPD participants were prescribed a single-inhaler triple therapy.

**Table 7:** Respiratory medicines prescribed in the 12 months prior to Ext-SLS consent, as recorded in primary care HER.

	Asthma (*N* = 798)	COPD (*N* = 349)
**Treatment,** ***n*** **(%)**		
SABA	662 (83.0)	314 (90.0)
SAMA	4 (<1)	15 (4.3)
SABA/SAMA (fixed-dose combination)	0	2 (<1)
SABD	663 (83.1)	315 (90.3)
ICS alone (single inhaler)	170 (21.3)	32 (9.3)
LABA alone (single inhaler)	41 (5.1)	33 (9.5)
LAMA alone (single inhaler)	59 (7.4)	250 (71.6)
ICS/LABA (FDC)	619 (77.6)	306 (87.7)
LABA/LAMA (FDC)	2 (<1)	32 (9.2)
ICS/LABA/LAMA (single inhaler)	27 (3.4)	87 (24.9)
LTRA	122 (15.3)	14 (4.0)
Methylxanthine	3 (<1)	8 (2.3)
PD4, theophylline	1 (<1)	4 (1.2)
Chronic OCS use^a^	21 (2.6)	27 (7.7)

*COPD* chronic obstructive pulmonary disease, *EHR* electronic health record, *Ext-SLS* extended Salford Lung Study, *FDC* fixed-dose combination, *ICS* inhaled corticosteroid, *LABA* long-acting β_2_-agonist, *LAMA* long-acting muscarinic antagonist, *LTRA* leukotriene receptor antagonists, *OCS* oral corticosteroid, *PD4* phosphodiesterase 4, *SABA* short-acting β_2_-agonist, *SABD* short-acting bronchodilator, *SAMA* short-acting muscarinic antagonist, *SD* standard deviation, *SLS* Salford Lung Study.

^a^Chronic OCS use defined as at least 4 courses with no gaps greater than 30 days; groups are not mutually exclusive.

### Description of participant-completed questionnaires

All participants completed some of the questionnaires, with most participants completing all required questions (asthma: 616 [77%]; COPD: 221 [63%]). The distribution of responses for each question was similar when analyzing the responses from all patients who completed the question compared to the subset of patients who completed all required questions (Tables S1,S2).

### Asthma

Among those who answered disease history questions (*n* = 738–755, variable number of responders), 31% reported the first occurrence of symptoms at age ≥40 years. The first diagnosis was made ≥40 years of age for 35% of patients, with 40% prescribed medication for the first time aged ≥40 years. Among the participants with asthma who responded to smoking questions (*n* = 784), 12% identified as current smokers (compared with 14% at SLS baseline and 14% as recorded in the primary care EHR data; Table 2).

**Fig. 1:**
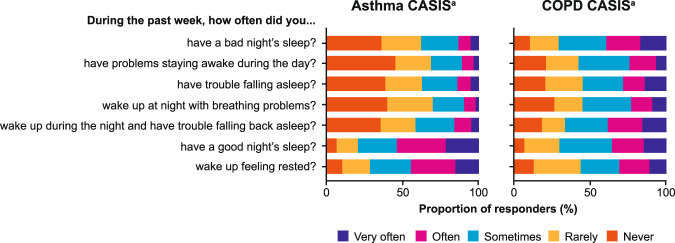
Disease impact on sleep among participants with asthma and COPD as reported at the time of Ext-SLS consent. CASIS COPD and Asthma Sleep Impact Scale, COPD chronic obstructive pulmonary disease, Ext-SLS extended Salford Lung Study. Note: ^a^One week look-back period for all seven questions. Asthma responders *n* = 783–792, COPD responders *n* = 335–343, variable per question.

The Asthma Control Test (ACT) was completed by 787 participants, 61% of whom scored ≥20 suggesting their asthma was well-controlled at the time of consent into the Ext-SLS. Scores suggesting partially controlled (ACT score 16–19) and uncontrolled (ACT score <16) asthma were found in 20 and 19% of participants, respectively. Of the 789 participants who completed the Asthma Control Questionnaire-6 (ACQ-6), 37% scored ≤0.75, indicating controlled asthma, while 31% scored >0.75 and <1.5 suggesting partial control and 32% scored ≥1.5 suggesting uncontrolled asthma. The effect of asthma on sleep, as measured by the COPD and Asthma Sleep Impact Scale (CASIS) total score, was found to be low among the Ext-SLS participants (*n* = 793), with 87% scoring <60 out of 100, placing them in the first tertile—indicating low impact (Fig. 1).

**Fig. 2:**
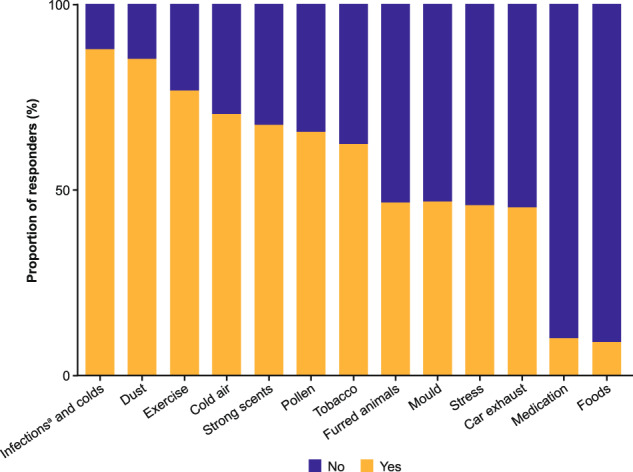
Common symptom triggers as reported by Ext-SLS participants with asthma at the time of Ext-SLS consent. Ext-SLS extended Salford Lung Study. Note: ^a^Of the airway. Responders providing a “Yes” or “No” answer *n* = 589–760 variable per question.

Commonly reported triggers for asthma symptoms were airway infections and colds (88%), dust (85%), exercise (77%), and cold air (70%) (Fig. 2). Alterations to maintenance medication dose or frequency were made without GP input by 25% of 783 participants, with 51% doing this ≥3 times in the preceding six months. The majority of responders had never been prescribed an oral corticosteroid rescue pack (82% of 759 participants) nor received a written asthma management plan (67% of 717 participants).

### COPD

**Fig. 3:**
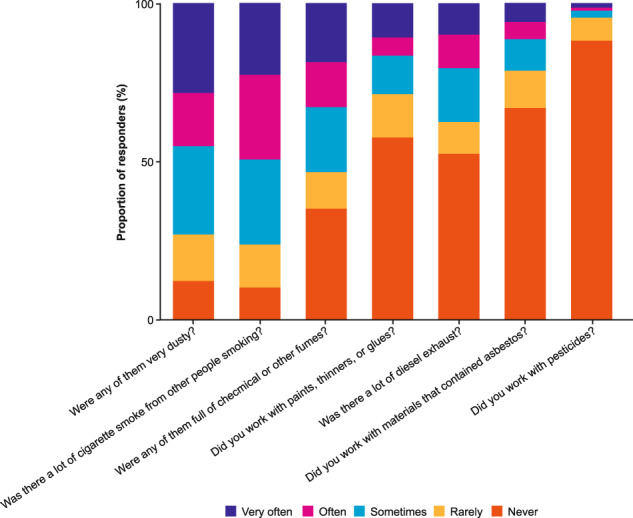
Self-reported working environments for participants with COPD in the Ext-SLS who reported working outside the home. COPD chronic obstructive pulmonary disease, Ext-SLS Extended Salford Lung Study. Note: Responders *n* = 337–346 variable per question.

A large proportion of responders (87% of 340) reported early exposure to second-hand tobacco smoke. For 66% of 342 participants, their mother was a smoker (Table S2). Among the participants with COPD who responded to smoking questions, 38% were classified as current smokers (compared with 46% at SLS baseline and 38% in the primary care EHR data). Among the participants who had ever smoked, 60% reported beginning smoking on most days between the ages of 15 and 19 years. When asked about environmental exposures, in the 93% of participants who had ever been employed, common exposures in the workplace were dust, cigarette smoke and fumes (including chemical fumes) (Fig. 3).

The mean (SD) COPD Assessment Test (CAT) score, among participants with COPD who completed this part of the questionnaire (*n* = 333), was 22.7 (8.7). Overall, 61% of participants scored >20, denoting a high impact of disease; the largest proportion of participants scored 21–30 (40%)—with lower scores denoting less disease burden. Based on the CASIS, most responders (*n* = 343) reported the little effect of their disease on sleep, with the majority (67%) scoring <60 out of 100 (first tertile, low impact on sleep) (Fig. 1). Among the other participants, 13% scored 60–70 (second tertile) and 20% scored ≥71 (third tertile). Most responders (37%) scored 1 on the modified Medical Research Council (mMRC) dyspnea scale, followed by 2 (29%).

In response to questions on social and physical functioning (*n* = 331–346, variable number of responders), 76% of participants reported visiting friends or family at least once per week; while 36 and 37% described being moderately satisfied and not at all satisfied with their health, respectively. Overall, 36% of participants reported feeling tired nearly every day, 16% on more than half of the days and 34% on several days in the two weeks prior to answering the questionnaire. Pub or social club attendance was reported by 29% of participants, 11% attended a sports club, gym, or golf club, 14% attended other group activities at least once per week but 53% reported that they did not attend any of the activities listed in the questionnaire. Being unable to walk unaided was described by 33% of participants, with COPD being a factor in the need for assistance in 88%.

## Discussion

The Ext-SLS offers a unique cohort of asthma and COPD patients who have been well characterized through involvement in a clinical trial, via EHR data collection and through completion of disease-specific questionnaires. Participants were originally recruited to the SLS after the presentation to primary care^12^ and are thus more representative of a real-world population than patients from traditional clinical trials.

Comparisons of the Ext-SLS cohort with the SLS ITT populations using SLS trial data demonstrated that the Ext-SLS cohort is broadly similar to the wider SLS populations, but with some key differences. Ext-SLS asthma participants were, on average older, with fewer symptoms than the SLS asthma ITT population at the time of SLS entry. Conversely, Ext-SLS participants with COPD were, on average, younger, but similarly had less severe disease (based on airflow obstruction) at the time of SLS entry than the SLS COPD ITT population. Additionally, it is likely that the benefits of randomization have not been carried through to the Ext-SLS, even if the 1:1 ratio of FF/VI to UC participants was broadly maintained. Interestingly, a previous observational cohort study compared the cohort of SLS COPD patients with matched non-trial patients with COPD in England from the Clinical Practice Research Datalink database^12^. The study found that the trial population was similar to the non-trial COPD population in terms of baseline demographics, clinical and treatment variables, including COPD exacerbations, supporting the generalizability of SLS COPD results. There was evidence of a Hawthorne effect (a phenomenon whereby participants or practitioners modify their behavior due to an awareness of being observed)^13,14^, as more COPD exacerbations were reported in trial patients and/or were more likely to be recorded. However, the largest effect was observed through changes in behavior in patients and general practitioner coding practices.

While the Ext-SLS may not fully represent the SLS, it remains a detailed and valuable cohort for respiratory disease study. The comprehensive primary care data available describe participants’ general health over the course of their disease, with measurements of BMI, laboratory parameters and information on comorbidities. The laboratory data provide insight into clinical biomarkers of respiratory disease, such as blood eosinophils and neutrophils, which are both potential biomarkers of disease severity^15–19^. Information on biomarkers, in combination with patient-reported information from the questionnaire, makes the Ext-SLS particularly useful in identifying disease traits (e.g., eosinophilic inflammation, smoking) and may help to address questions relating to the impact of a treatable traits^6,20^ approach (in contrast to stepwise treatment approaches as are routinely used in asthma management^18^) in obstructive airways disease.

Additionally, data on the prescription of respiratory medication can indicate common treatment patterns and indirectly provide information on disease control. A noteworthy limitation of the prescribing data is the restriction to prescriptions issued in primary care. Most new and repeat prescriptions for respiratory medications are issued in primary care, except biological therapies for asthma. Additionally, the exact dates of prescriptions were not available at the time of analysis (only month and year), meaning that we could not accurately determine the use of multiple-inhaler triple therapies.

The questionnaires (completed in full by a large proportion of participants) complement the EHR data, providing information on disease impact that is not typically available. Among Ext-SLS participants with asthma, the validated ACT, ACQ, and CASIS questionnaires detailed the Ext-SLS asthma population as one where many participants still have uncontrolled asthma, and disturbed sleep, albeit at a low level. The questionnaires also showed a high level of self-management among the participants.

Participants with COPD in the Ext-SLS had a mean CAT score >20, suggesting that COPD had a substantial impact on the lives of these participants. However, as with the asthma population, the CASIS results suggested that most participants did not find their sleep suffering as a result of their COPD, though many reported sleep disturbances. The social impact of COPD was demonstrated by the questionnaires, with more than half of participants reporting that they did not attend social activities—aligning with previous research into the social impact of COPD^21^. Data relating to early life and personal history provided valuable insights that could not be gathered from medical records, detailing factors relating to underlying etiologies. The asthma questionnaires indicated that asthma was often adult-onset in this cohort and outlined common triggers of symptoms. For participants with COPD, data from the questionnaires suggested factors that could have contributed to the disease in the household and working environments.

The Ext-SLS has some limitations that relate to the nature of the data. Patient-reported outcomes were collected in the disease-specific questionnaires and could be susceptible to inaccuracy or error due to factors such as limitations in recall, recall bias and a subjective assessment of the variables. However, validated instruments were used to collect information about disease impact and management^22–28^. Similarly, the nature of real-world data means that data may be error-prone (e.g., inconsistent data recording and missing data). The time between the original SLS and the Ext-SLS led to patient attrition and there may be bias in the recruitment of patients due to the SLS population ageing and possibly becoming too unwell to participate.

In conclusion, the Ext-SLS cohort is a well-characterized and valuable resource in the field of respiratory research and may be particularly useful in addressing questions relating to treatable traits. The cohort is unique due to the inclusion of a randomized clinical trial in its timeline. Ongoing work collecting prospective data from the cohort will further increase the data available over time.

## Supplementary information

Supplementary material

Reporting Summary

## Data Availability

Anonymized individual participant data and study documents can be requested for further research from www.clinicalstudydatarequest.com. The secondary care data associated with this study are under license from NHS Digital and cannot be released as such.
