# Supplementary material for: Extending the data collection from a clinical trial: The Extended Salford Lung Study research cohort
Source: NPJ Prim Care Respir Med. 2023 Jan 18;33:4. doi: 10.1038/s41533-022-00322-7 (PMC9845305; doi:10.1038/s41533-022-00322-7)
Supplement: Supplementary file 1 — Supplementary material [file 41533_2022_322_MOESM1_ESM.pdf]

Supplementary material

Supplementary Table 1. Asthma questionnaire responses from the Known* and Complete† sub-groups

Questions	Ranges	Patients with Known Responses*		Patients with Complete Questionnaire Data†	
		(N=variable)	%	(N=616)	%
How old were you when you first got asthma or had symptoms of asthma?	**Age at first asthma symptoms**	***751***	***94.11***	***616***	***77.19***
	Birth to 1 year	43	5.73	39	6.33
	1 to 4 years	59	7.86	51	8.28
	5 to 9 years	88	11.72	71	11.53
	10 to 14 years	73	9.72	61	9.9
	15 to 19 years	59	7.86	48	7.79
	20 to 29 years	98	13.05	75	12.18
	30 to 39 years	97	12.92	74	12.01
	40 years or older	234	31.16	169	27.44
	Don't Know			28	4.55
How old were you when you were first told by a doctor, nurse, or other health professional that you had asthma?	**Age at first asthma diagnosis**	***755***	***94.61***	***616***	***77.19***
	Birth to 1 year	24	3.18	21	3.41
	1 to 4 years	55	7.28	50	8.12
	5 to 9 years	73	9.67	63	10.23
	10 to 14 years	60	7.95	52	8.44
	15 to 19 years	63	8.34	51	8.28
	20 to 29 years	110	14.57	86	13.96
	30 to 39 years	104	13.77	78	12.66
	40 years or older	266	35.23	193	31.33
	Don't Know			22	3.57
How old were you when you were prescribed daily preventative asthma medication (i.e. a preventer inhaler), other than a reliever medication (in a blue inhaler e.g. salbutamol or terbutaline§)?	**Age at first daily preventative asthma medication**	**738**	**92.48**	**616**	**77.19**
	Birth to 1 year	6	0.81	5	0.81
	1 to 4 years	34	4.61	30	4.87
	5 to 9 years	53	7.18	48	7.79
	10 to 14 years	50	6.78	42	6.82
	15 to 19 years	59	7.99	48	7.79
	20 to 29 years	126	17.07	105	17.05
	30 to 39 years	116	15.72	85	13.8
	40 years or older	294	39.84	214	34.74
	Don't Know			39	6.33
	**ACQ Components**				
Summary score	**ACQ Score**	**789**	**98.87**	**616**	**77.19**
	Mean (SD)	1.29	1.04	1.25	1.01
	Median (IQR)	1	(0.5, 1.83)	1	(0.5, 1.67)

	Min, Max	0	5.5	0	5
ACQ category	**ACQ Category**	**789**	**98.87**	**616**	**77.19**
	well-controlled (ACQ <0.75)	290	36.76	232	37.66
	adequate control (0.75-1.5)	244	30.93	194	31.49
	poor control (ACQ >1.5)	255	32.32	190	30.84
	**Triggers**				
Furred animals, for example: dogs, cats, cows, horses, rabbits, etc	**Animal**	**717**	**89.85**	**616**	**77.19**
	Yes	336	46.86	288	46.75
	No	381	53.14	284	46.10
	Don't Know			44	7.14
Pollen exposure, for example: leaves, grass, outdoor flowers	**Pollen**	**734**	**91.98**	**616**	**77.19**
	Yes	483	65.8	380	61.69
	No	251	34.2	202	32.79
	Don't Know			34	5.52
Smell of mould or exposure to mould	**Mould**	**589**	**73.81**	**616**	**77.19**
	Yes	276	46.86	212	34.42
	No	313	53.14	257	41.72
	Don't Know			147	23.86
Tobacco smoke or smell of tobacco	**Tobacco**	**729**	**91.35**	**616**	**77.19**
	Yes	454	62.28	349	56.66
	No	275	37.72	233	37.82
	Don't Know			34	5.52
Dusty places	**Dust**	**744**	**93.23**	**616**	**77.19**
	Yes	636	85.48	496	80.52
	No	108	14.52	90	14.61
	Don't Know			30	4.87
Strong-smelling scents	**Strong scents**	**751**	**94.11**	**616**	**77.19**
	Yes	507	67.51	388	62.99
	No	244	32.49	202	32.79
	Don't Know			26	4.22
Car exhaust	**Car exhaust**	**674**	**84.46**	**616**	**77.19**
	Yes	305	45.25	239	38.8
	No	369	54.75	295	47.89
	Don't Know			82	13.31
Air pollution, other than car exhaust	**Other air pollution**	**601**	**75.31**	**616**	**77.19**
	Yes	284	47.25	218	35.39
	No	317	52.75	264	42.86
	Don't Know			134	21.75
Airway infections or colds	**Airway infections or colds**	**760**	**95.24**	**616**	**77.19**
	Yes	669	88.03	520	84.42
	No	91	11.97	79	12.82
	Don't Know			17	2.76
	**Medications**	**640**	**80.20**	**616**	**77.19**

Medicines, for example: aspirin	Yes	64	10	51	8.28
	No	576	90	467	75.81
	Don't Know			98	15.91
Food, for example: fish, shellfish, or nuts	**Foods**	**709**	**88.85**	**616**	**77.19**
	Yes	63	8.89	46	7.47
	No	646	91.11	522	84.74
	Don't Know			48	7.79
Psychological factors or stress	**Stress**	**706**	**88.47**	**616**	**77.19**
	Yes	324	45.89	241	39.12
	No	382	54.11	318	51.62
	Don't Know			57	9.25
Cold air	**Cold air**	**755**	**94.61**	**616**	**77.19**
	Yes	531	70.33	412	66.88
	No	224	29.67	182	29.55
	Don't Know			22	3.57
Exercise or taking part in sports	**Exercise**	**746**	**93.48**	**616**	**77.19**
	Yes	573	76.81	447	72.56
	No	173	23.19	139	22.56
	Don't Know			30	4.87
	**Medical History**				
Do you ever change the dose or frequency of your preventer inhaler without your doctor or nurse instructing you to do so?	**Medication dose or frequency change without GP instruction**	**783**	**98.12**	**616**	**77.19**
	Yes	192	24.52	154	25
	No	591	75.48	460	74.68
	Don't Know			2	0.32
How many times in the past six months has this occurred?	**Medication change in last 6 months**	**779**	**97.62**	**616**	**77.19**
	0 times	6	3.28‡	4	2.60‡
	1-2 times	84	45.90	71	46.10
	3-4 times	43	23.50	34	22.08
	5-6 times	20	10.93	14	9.09
	7 or more times	30	16.39	25	16.23
	Don't Know			6	3.90
	Skipped	596	NA	462	NA
Has your doctor or nurse prescribed steroid tablets (a "rescue pack") to keep at home for use if your asthma symptoms worsen?	**Ever prescribed OCS Rescue packs**	**759**	**95.11**	**616**	**77.19**
	Yes	139	18.31	107	17.37
	No	620	81.69	499	81.01
	Don't Know			10	1.62
Has your doctor or nurse given you a written asthma management plan?	**Written asthma management plan in place**	**717**	**89.85**	**616**	**77.19**
	Yes	238	33.19	191	31.01
	No	479	66.81	393	63.8
	Don't Know			32	5.19

Summary Score	**CASIS Summary Score**	**793**	**99.37**	**616**	**77.19**
	With score (≤3 missing), N (%)	793	100	616	100
	Mean (SD)	32.21	22.53	31.59	22.64
	Median (IQR)	32.14	(10.71, 46.43)	28.57	(10.71, 46.43)
	Min, Max	0	100	0	100
CASIS Categories	**CASIS Categories**	**793**	**99.37**	**616**	**77.19**
	Tertile-1: CASIS <60	690	87.01	538	87.34
	Tertile-2: CASIS 60-<71	42	5.3	28	4.55
	Tertile-3: CASIS ≤71	61	7.69	50	8.12
Smoking Exposure	**Smoking Status**	**784**	**98.25**	**616**	**77.19**
	Current smoker	91	11.61	70	11.38
	Former smoker	316	40.31	249	40.49
	Never smoker	377	48.09	296	48.13
	Don't Know			***1***	***NA***
Summary Score	**ACT Summary Score**	**787**	**98.62**	**616**	**77.19**
	Mean (SD)	19.67	4.37	19.69	4.37
	Median (IQR)	21	(17, 23)	21	(17, 23)
	Min, Max	6	25	6	25
ACT Category	**ACT Category**	**787**	**98.62**	**616**	**77.19**
	Well controlled, ≥20	483	61.37	382	62.01
	Partial control, 16 – <20	156	19.82	120	19.48
	Uncontrolled, <16	148	18.81	114	18.51

*Including any answers given other than "Don't Know"; †Including answers only from participants who answered all applicable questions fully; ‡Percentage based on un-skipped total; §brand names used in actual questionnaire.

ACQ, Asthma Control Questionnaire; ACT, Asthma Control Test; CASIS, COPD and Asthma Sleep Impact Scale; GP, general practitioner; IQR, interquartile range; OCS, oral corticosteroid; SABA, short-acting beta-agonist; SD, standard deviation

Supplementary Table 2. COPD questionnaire responses from the Known* and Complete† sub-groups

Questions	Ranges	Known Data*		Complete Data†	
		N	%	N	%
Did either of your parents, or another adult in your household, smoke regularly around you during childhood?	**Childhood exposure to smoke**	**340**	**97.42**	**221**	**63.32**
	Yes	296	87.06	186	84.16
	No	44	12.94	30	13.57
	Don't Know			5	2.26
Was it your mother who smoked?	**Maternal smoking**	**342**	**97.99**	**221**	**63.32**
	Yes	191	65.86	112	60.22
	No	99	34.14	73	39.25
	Don't Know			1	0.54
	Skipped	52	NA	35	NA
Smoking summary	**Smoking Status**	**325**	**85.96**	**221**	**63.32**
	Current smoker	113	37.67	57	28.64
	Ex-smoker	183	61	139	69.85
	Never smoker	4	1.33	3	1.51
	Skipped	25	NA	22	NA
	**Occupational and Environmental exposures**				
Have you ever been employed (i.e. worked outside of the home?)	**Employed outside the home**	**340**	**97.42**	**221**	**63.32**
	Yes	317	93.24	208	94.12
	No	23	6.76	13	5.88
Thinking about the places where you worked…	**Were any of them very dusty?**	**344**	**98.57**	**221**	**63.32**
	Never	40	12.46	25	12.02
	Rarely	47	14.64	34	16.35
	Sometimes	89	27.73	58	27.88
	Often	55	17.13	37	17.79
	Very often	90	28.04	54	25.96
	Skipped	23	NA	13	NA
Were any of them full of chemical or other fumes?	**Were any of them full of chemical or other fumes?**	**340**	**97.42**	**221**	**63.32**
	Never	112	35.33	76	36.54
	Rarely	36	11.36	18	8.65
	Sometimes	65	20.50	46	22.12
	Often	45	14.20	29	13.94
	Very often	59	18.61	39	18.75
	Skipped	23	NA	13	NA
Was there a lot of cigarette smoke from other people smoking?	**Was there a lot of cigarette smoke from other people smoking?**	**340**	**97.42**	**221**	**63.32**
	Never	32	10.09	21	10.10
	Rarely	44	13.88	30	14.42

	Sometimes	85	26.81	57	27.40
	Often	84	26.50	56	26.92
	Very often	72	22.71	44	21.15
	Skipped	23	NA	13	NA
Did you work with materials that contained asbestos?	**Did you work with materials that contained asbestos?**	**337**	**96.56**	**221**	**63.32**
	Never	210	66.88	142	68.27
	Rarely	38	12.10	23	11.06
	Sometimes	31	9.87	23	11.06
	Often	17	5.41	10	4.81
	Very often	18	5.73	10	4.81
	Skipped	23	NA	13	NA
Did you work with paints, thinners, or glues?	**Did you work with paints, thinners, or glues?**	**342**	**97.99**	**221**	**63.32**
	Never	185	57.99	124	59.62
	Rarely	42	13.17	26	12.50
	Sometimes	40	12.54	27	12.98
	Often	18	5.64	8	3.85
	Very often	34	10.66	23	11.06
	Skipped	23	NA	13	NA
Did you work with pesticides?	**Did you work with pesticides?**	**341**	**97.71**	**221**	**63.32**
	Never	281	88.36	188	90.38
	Rarely	23	7.23	13	6.25
	Sometimes	8	2.52	4	1.92
	Often	3	0.94		0.00
	Very often	3	0.94	3	1.44
	Skipped	23	NA	13	NA
Was there a lot of diesel exhaust?	**Was there a lot of diesel exhaust?**	**346**	**99.14**	**221**	**63.32**
	Never	170	52.63	109	52.40
	Rarely	32	9.91	19	9.13
	Sometimes	55	17.03	39	18.75
	Often	34	10.53	21	10.10
	Very often	32	9.91	20	9.62
	Skipped	23	NA	13	NA
	**Social Support and Physical Functioning**				
How often do you visit friends or family or have them visit you?	**Social Visits**	**346**	**99.14**	**221**	**63.32**
	Almost daily	81	23.41	50	22.62
	2 – 4 times a week	96	27.75	63	28.51
	About once a week	86	24.86	49	22.17
	About once a month	42	12.14	30	13.57
	Once every few months	26	7.51	20	9.05
	Never or almost never	12	3.47	6	2.71

	No friends or family outside of the household	3	0.87	1	0.45
	Don't Know			2	0.9
Which of the following do you attend once a week or more often?	**Social Activities**	**344**	**98.57**	**221**	**63.32**
	Sports club, gym, or golf club	36	10.47	27	12.22
	Religious group	19	5.52	13	5.88
	Pub or social club	99	28.78	63	28.51
	Adult education class	2	0.58	1	0.45
	Other group activity	47	13.66	35	15.84
	None of these	183	53.2	115	52.04
In general how satisfied are you with your health?	**Health Status**	**344**	**98.57**	**221**	**63.32**
	Extremely satisfied	1	0.29	1	0.45
	Moderately Satisfied	124	36.05	79	35.75
	Satisfied	47	13.66	36	16.29
	Somewhat satisfied	45	13.08	27	12.22
	Not satisfied at all	127	36.92	77	34.84
	Don't Know			1	0.45
Do you often feel lonely?	**Loneliness**	**331**	**94.84**	**221**	**63.32**
	Yes	93	28.1	52	23.53
	No	238	71.9	160	72.4
	Don't Know			9	4.07
Over the past two weeks, how often have you felt tired or had little energy?	**Energy Level**	**337**	**96.56**	**221**	**63.32**
	Not at all	47	13.95	28	12.67
	Several days	114	33.83	78	35.29
	More than half the days	55	16.32	42	19
	Nearly every day	121	35.91	69	31.22
	Don't know			4	1.81
Are you able to walk by yourself without help from a cane, walking frame, or other person to help you?	**Walking without assistance**	**339**	**97.13**	**221**	**63.32**
	Yes	226	66.67	152	68.78
	No	113	33.33	68	30.77
	Don't Know			1	0.45
Do you need assistance walking as a result of your COPD or as a result of something else?	**Reason for needing walking assistance**	**341**	**97.71**	**221**	**63.32**
	COPD	36	32.14	20	29.41
	Something else	14	12.50	7	10.29
	COPD and something else	62	55.36	40	58.82
	Don't know			1	1.47
	Skipped	229	NA	153	NA
Which of the following best describes how breathless you get these days?	**Breathlessness**	**346**	**99.14**	**221**	**63.32**
	I only get breathless with strenuous exercise	34	9.83	24	10.86
	I get short of breath when hurrying on level ground or walking up a slight hill	129	37.28	86	38.91
	On level ground, I walk slower than other people of the same	100	28.9	62	28.05

	age because of breathlessness, or I have to stop for breath when walking at my own pace on the level				
	I stop for breath after walking about 100 yards or after a few minutes on level ground	57	16.47	34	15.38
	I am too breathless to leave the house or I am breathless when dressing	26	7.51	15	6.79
CASIS Summary Score	**CASIS Summary Score**	**343**	**98.28**	**221**	**63.32**
	Missing score (>3 missing), N (%)				
	With score (≤3 missing), N (%)	343	100%	221	100%
	Mean (SD)	47.22	24.38	45.3	23.89
	Median (IQR)	50	(28.57, 67.86)	46.43	(28.57, 64.29)
	Min, Max	0	100	0	96.43
CASIS Category	**CASIS Category**	**343**	**98.28**	**221**	**63.32**
	Tertile-1: CASIS < 60	231	67.35	155	70.14
	Tertile-2: CASIS 60-70	45	13.12	27	12.22
	Tertile-3: CASIS >=71	67	19.53	39	17.65
CAT Summary Score summary	**CAT Summary Score**	**333**	**95.42**	**221**	**63.32**
	Mean (SD)	22.68	8.68	21.67	8.57
	Median (IQR)	24	(16, 30)	22	(15, 28)
	Min, Max	3	40	3	40
CAT Summary Score Range	0 – 10	39	11.71	26	11.76
	11 – 20	90	27.03	73	33.03
	21 – 30	133	39.94	84	38.01
	31 – 40	71	21.32	38	17.19
CAT Summary Score Category	≤20	129	38.74	99	44.8
	≥21	204	61.26	122	55.2

*Including any answers given other than "Don't Know"; †Including answers only from participants who answered all applicable questions fully; ‡Percentage based on un-skipped total.

CASIS, COPD and Asthma Sleep Impact Scale; CAT, COPD Assessment Test; COPD, chronic obstructive pulmonary disease; IQR, interquartile range; SD, standard deviation.

Supplementary File 1. Asthma-specific questionnaire provided to patients in the informed consent packages

Asthma Questionnaire

This questionnaire is a collection of mini surveys: we will ask you some questions about your asthma diagnosis, symptoms, and triggers for your asthma, as well as how you manage it. You will also be asked to complete the **Asthma Control Questionnaire**, the **Asthma Control Test** and a short sleep survey.

The whole questionnaire should take you approximately 20–25 minutes to complete.

Please answer honestly and as best as you can. If you don't know the answer or prefer not to answer a particular question, please select 'Don't know/No answer'. If you cannot find an exact answer, please select the closest response.

Please be reassured that all of your answers will be treated with absolute confidentiality. If you have any questions about how your answers will be used or if you have any difficulties with completing this, please contact the patient recruitment company overseeing the project, Ignite Data (Phone: 0800 368 9915 Mon-Fri 9am - 5pm, quoting "Ex-SLS.

Instructions are shown as bold orange text.

Your Asthma History

H1 How old were you when you first got asthma or had symptoms of asthma?

☐ From birth to 1 year	☐ 1–4	☐ 5–9
☐ 10–14	☐ 15–19	☐ 20–29
☐ 30–39	☐ 40 years or older	☐ Don’t know/No answer

H2 How old were you when you were first told by a doctor, nurse or other health professional that you had asthma?

☐ Less than 1 year	☐ 1–4	☐ 5–9
☐ 10–14	☐ 15–19	☐ 20–29
☐ 30–39	☐ 40 years or older	☐ Don’t know/No answer

H3 How old were you when you were prescribed daily preventive asthma medication (i.e. a preventer inhaler), other than a reliever medication (in a blue inhaler e.g. Ventolin or Bricanyl)?

☐ Less than 1 year	☐ 1–4	☐ 5–9
☐ 10–14	☐ 15–19	☐ 20–29
☐ 30–39	☐ 40 years or older	☐ Don’t know/No answer

Asthma Control Questionnaire

The next section looks a bit different. It consists of questions from the Asthma Control Questionnaire (ACQ). Please follow the instructions on the next page.

PRO Redacted

PRO Redacted

PRO Redacted

Triggers for Your Asthma

Thinking of when you have asthma symptoms, what factors provoke wheezing or whistling in the chest, attacks of shortness of breath or periods of breathlessness, or cough:

		Yes	No	Don't know/ No Answer
T1	Furred animals, for example: dogs, cats, cows, horses, rabbits etc?	☐	☐	☐
T2	Pollen exposure, for example: leaves, grass, outdoor flowers?	☐	☐	☐
T3	Smell of mould or exposure to mould?	☐	☐	☐
T4	Tobacco smoke or smell of tobacco?	☐	☐	☐
T5	Dusty places?	☐	☐	☐
T6	Strong smelling scents, for example: perfume, spices, printing ink, smell of frying, cleaning products, strong smelling flowers etc?	☐	☐	☐
T7	Car exhausts?	☐	☐	☐
T8	Air pollution, other than car exhausts?	☐	☐	☐
T9	Airway infections or colds?	☐	☐	☐
T10	Medicines, for example: aspirin?	☐	☐	☐
T11	Food, for example: fish, shellfish, or nuts?	☐	☐	☐
T12	Psychological factors or stress?	☐	☐	☐
T13	Cold air?	☐	☐	☐
T14	Exercise or taking part in sports?	☐	☐	☐

Asthma Management

M1	Do you ever change the dose or frequency of your preventer inhaler for asthma (e.g. Seretide, Symbicort, Flixotide, Qvar, Pulmicort) without your doctor or nurse instructing you to do so?<br><br>**Please do not consider changes in the use of your reliever inhaler (a blue inhaler e.g. Ventolin or Bricanyl) for your response.**	☐ Yes	**Go to M2**
		☐ No	**Skip to M3**
		☐ Don't know/No answer	**Skip to M3**

M2 How many times in the past **six months** has this occurred?

☐ 0	☐ 1–2	☐ 3–4
☐ 5–6	☐ 7 or more	☐ Don't know/ No answer

M3	Has your doctor or nurse prescribed steroid tablets (a "rescue pack") to keep at home for use if your asthma symptoms worsen?	☐ Yes<br>☐ No<br>☐ Don't know/No answer
M4	Has your doctor or nurse given you a <u>written</u> asthma management plan (i.e. a plan for what medication you take every day, what you do when you feel worse, and what you should do in an asthma attack)?	☐ Yes<br>☐ No<br>☐ Don't know/No answer

Asthma Management

M5	Some patients use **more** puffs/inhalations of their reliever inhaler (a blue inhaler e.g. Ventolin or Bricanyl) when they have an ‘asthma attack’ that **does not** require either A&E/hospital attendance or use of a steroid rescue pack.<br><br>In your mind, how many **extra** puffs/inhalations would you have to use in one day to say that you are having this kind of an ‘asthma attack’?	☐	I don’t use **more** reliever inhaler	Skip to the next section (S1)
		☐	1 **extra** puff	Go to M6
		☐	2 **extra** puffs	Go to M6
		☐	3 **extra** puffs	Go to M6
		☐	4 **extra** puffs	Go to M6
		☐	5 or more **extra** puffs	Go to M6
		☐	Don’t know/No answer	Skip to the next section (S1)
M6	How many times has this occurred in the past **year**?	☐	0	
		☐	1–3	
		☐	4–6	
		☐	7–9	
		☐	10 or more	
		☐	Don’t know/No answer	

Asthma Impact on Sleep

PRO Redacted

Exposure to Tobacco

We would like you to tell us about your smoking history. We value your honest opinion on the following questions.

	Question	Response	Routing
E1	Do you think of yourself as a tobacco 'smoker'?	☐ Yes	**Skip to E4**
		☐ No	**Go to E2**
		☐ Don't know/No answer	**Go to E2**
E2	Have you ever smoked tobacco?	☐ Yes	**Go to E3**
		☐ No	**Skip to the next section**
		☐ Don't know/No answer	**Go to E3**
E3	In your lifetime, have you smoked a total of at least 100* times?<br>** 5 normal-sized packs is 100 cigarettes, 10 cigarettes per year over 10 years is also 100 cigarettes*	☐ Yes	**Go to E4**
		☐ No	**Skip to the next section**
		☐ Don't know/No answer	**Skip to the next section**
E4	Have you ever smoked anything other than cigarettes, for example a pipe, cigar, electronic cigarette, marijuana or shisha?	☐ Yes	
		☐ No	
		☐ Don't know/No answer	

Asthma Control Test

The next section looks a bit different. It consists of questions from the Asthma Control Test. Please follow the instructions on the next page.

[redacted]

PRO Redacted

Last Questions

L1 Date you completed this questionnaire:

____ / ____ / _____

DD **MM** **YYYY**

L2 Are you currently participating in a clinical trial for your asthma, not including the Salford Lung Study (SLS)?

☐ Yes ☐ No ☐ Don't know/ No answer

L3 Have you participated in a clinical trial for your asthma since the Salford Lung Study?

☐ Yes ☐ No ☐ Don't know/ No answer

Thank you for completing this questionnaire!

Supplementary File 2. COPD-specific questionnaire provided to patients in the informed consent packages

COPD Questionnaire

This questionnaire is a collection of mini surveys: we will ask you some questions about factors which may have contributed to your COPD. These may have been experienced over your lifetime in your home or workplace and include exposure to tobacco. We will also ask about your early life experience and possible history of respiratory diseases among your family members. You will also be asked to complete the **COPD Assessment Test** and a short sleep survey.

The whole questionnaire should take you approximately 25–30 minutes to complete.

Please answer honestly and as best as you can. If you don't know the answer or prefer not to answer a particular question, please select 'Don't know/No answer'. If you cannot find an exact answer, please select the closest response.

Please be reassured that all of your answers will be treated with absolute confidentiality. If you have any questions about how your answers will be used or if you have any difficulties with completing this, please contact the patient recruitment company overseeing the project, Ignite Data (Phone: 0800 368 9915 Mon-Fri 9am - 5pm, quoting "Ex-SLS").

Instructions are shown as bold orange text.

Your Birth and Early-Life Experiences

E1 How old was your mother when you were born?

- ☐ Less than 20 years
- ☐ 20–24
- ☐ 25–29
- ☐ 30–34
- ☐ 35–39
- ☐ 40 years or older
- ☐ Don't know/No answer

E2 Were you a twin, triplet or other multiple birth?

- ☐ Yes
- ☐ No
- ☐ Don't know/No answer

E3 Were you born prematurely (before 37 weeks of pregnancy)?

- ☐ Yes
- ☐ No
- ☐ Don't know/No answer

E4 Were you hospitalised before the age of two years for lung disease?

- ☐ Yes
- ☐ No
- ☐ Don't know/No answer

E5 What term best describes the place* you lived most of the time when you were under the age of 5 years?

**house or immediate surroundings*

Select one answer only.

- ☐ Farm
- ☐ Village or small town in a rural area
- ☐ Medium to large city
- ☐ Don't know/No answer

Your Birth and Early-Life Experiences

E6	When you were 10 years old, compared to average children your age, would you describe yourself as:	☐ Thinner	
		☐ Plumper/heavier	
		☐ About the same	
		☐ Don’t know/No answer	
E7	Did either of your parents, or another adult in your household, smoke regularly around you during your childhood?	☐ Yes	**Go to E8**
		☐ No	**Skip to the next section (T1)**
		☐ Don’t know/No answer	**Skip to the next section (T1)**
E8	If yes, was it your mother who smoked?	☐ Yes	
		☐ No	
		☐ Don’t know/No answer	

Exposure to Tobacco or Second-hand Smoke

We would like you to tell us about your smoking history. We value your honest opinion on the following questions.

T1	Does anyone in your current household smoke (indoors or outdoors, **not including you**)?	☐ Yes, one household member smokes	
		☐ Yes, more than one household member smokes	
		☐ No, no one smokes	
T2	Do you think of yourself as a tobacco 'smoker'?	☐ Yes	**Skip to T5**
		☐ No	**Go to T3**
		☐ Don't know/No answer	**Go to T3**
T3	Have you ever smoked tobacco?	☐ Yes	**Go to T4**
		☐ No	**Skip to the next section (F1)**
		☐ Don't know/No answer	**Go to T4**

Exposure to Tobacco or Second-hand Smoke

T4	In your lifetime, have you smoked a total of at least 100* times?<br><br>** 5 normal-sized packs is 100 cigarettes, 10 cigarettes per year over 10 years is also 100 cigarettes*	☐ Yes	**Go to T5**
		☐ No	**Skip to the next section (F1)**
		☐ Don't know/No answer	**Skip to the next section (F1)**
T5	Have you ever smoked anything other than cigarettes, for example a pipe, cigar, electronic cigarette, marijuana or shisha?	☐ Yes	
		☐ No	
		☐ Don't know/No answer	

T6 How old were you when you first started smoking on most days?

☐ Less than 10 years	☐ 10–14	☐ 15–19	☐ 20–24
☐ 25–29	☐ 30–39	☐ 40 years or older	☐ Don't know/ No answer

Only answer questions T7 and T8 if you answered 'Yes' to question T2, otherwise skip to T9.

T7	Have you ever tried to quit smoking in the past?	☐ Yes	**Go to T8**
		☐ No, I have never tried to quit	**Skip to the next section (F1)**
		☐ Don't know/ No answer	

Exposure to Tobacco or Second-hand Smoke

T8 During your quit attempt(s), for how long were you not a regular smoker?

_______ years

______ months

_______ weeks

Only answer question T9 if you answered 'Yes' to questions T3 and T4, otherwise skip to the next section.

T9 What age were you when you last smoked regularly??

- ☐ Less than 20 years
- ☐ 20–29
- ☐ 30–39
- ☐ 40–49
- ☐ 50–59
- ☐ 60 years or older
- ☐ Don't know/No answer

Respiratory Disease in Your Family

F1 Was your **father** or **mother** ever diagnosed with or did he/she suffer from any of the following?

It's OK if you don't know. You can select more than one answer.

- ☐ COPD, chronic bronchitis, emphysema
- ☐ Asthma
- ☐ Lung cancer
- ☐ None of the above
- ☐ Don't know/No answer

Occupational and Environmental Exposures

O1 Have you ever been employed (i.e. worked outside of the home)?

☐ Yes — **Go to O2**

☐ No — **Skip to O10**

Thinking about the places where you worked:

	Never	Rarely	Sometimes	Often	Very Often
O2. Were any of them very dusty?	☐	☐	☐	☐	☐
O3. Were any of them full of chemical or other fumes?	☐	☐	☐	☐	☐
O4. Was there a lot of cigarette smoke from other people smoking?	☐	☐	☐	☐	☐
O5. Did you work with materials that contained asbestos?	☐	☐	☐	☐	☐
O6. Did you work with paints, thinners or glues?	☐	☐	☐	☐	☐
O7. Did you work with pesticides?	☐	☐	☐	☐	☐
O8. Was there a lot of diesel exhaust?	☐	☐	☐	☐	☐

Occupational and Environmental Exposures

O9	When you were employed, did you have problems with your breathing that improved when you stopped working, went on holiday, or were away from your workplace?	☐ Yes<br>☐ No<br>☐ Don't know/No answer

O10 Over your lifetime, which type of hob did you or your family mostly use for cooking?

Please select one answer.

☐ A gas hob or gas cooker	☐ Electric or induction hob
☐ Solid fuel (coal, coke or wood)	☐ Other (i.e., oil [kerosene] paraffin)
☐ None of these	☐ Don't know/No answer

O11 Over your lifetime, which type of heating was mostly used to warm your home?

You can select more than one answer.

☐ Gas central heating	☐ Electric heaters
☐ Oil (kerosene) central heating	☐ Portable gas or paraffin heaters
☐ Solid fuel central heating	☐ Open fire
☐ None of these	☐ Don't know/No answer

Social Support and Physical Functioning

SS1	How often do you visit friends or family or have them visit you?<br>*(Visits can be a few minutes, a few hours or even overnight.)*	☐ Almost daily<br>☐ 2–4 times per week<br>☐ About once a week<br>☐ About once a month<br>☐ Once every few months<br>☐ Never or almost never<br>☐ No friends/family outside household<br>☐ Don't know/No answer

SS2 Which of the following do you attend once a week or more often?

You can select more than one answer.

☐ Sports club, gym or golf club	☐ Pub or social club
☐ Religious group	☐ Adult education class
☐ Other group activity	☐ None of these

SS3 In general, how satisfied are you with your health?

☐ Extremely satisfied	☐ Moderately satisfied
☐ Satisfied	☐ Somewhat satisfied
☐ Not satisfied at all	☐ Don't know/No answer

SS4	Do you **often** feel lonely?	☐ Yes<br>☐ No<br>☐ Don't know/No answer

Social Support and Physical Functioning

SS5 Over the **past two weeks**, how often have you felt tired or had little energy?

- ☐ Not at all
- ☐ Several days
- ☐ More than half the days
- ☐ Nearly every day
- ☐ Don't know/No answer

SS6 Are you able to walk by yourself without help from a cane, walking frame, or other person to help you?

- ☐ Yes — Skip to SS8
- ☐ No, I need some assistance to walk — Skip to SS7
- ☐ Don't know/No answer — Skip to SS8

SS7 Do you need assistance walking as a result of your COPD or as a result of something else?

- ☐ My COPD
- ☐ Something else
- ☐ Both my COPD and something else
- ☐ Don't know/No answer

Social Support and Physical Functioning

SS8 Which of the following best describes how breathless you get these days?

Please select only one answer.

- ☐ 1.) I only get breathless with strenuous exercise
- ☐ 2.) I get short of breath when hurrying on level ground or walking up a slight hill
- ☐ 3.) On level ground, I walk slower than people of the same age because of breathlessness, or I have to stop for breath when walking at my own pace on the level
- ☐ 4.) I stop for breath after walking about 100 yards or after a few minutes on level ground
- ☐ 5.) I am too breathless to leave the house or I am breathless when dressing

COPD Impact on Sleep

[redacted]

PRO Redacted

COPD Assessment Test

The next section looks a bit different. It consists of questions from the COPD Assessment Test. Please follow the instructions on the next page.

PRO Redacted

Last Questions

L1 Date you completed this questionnaire:

____ / ____ / _____

DD MM YYYY

L2 Are you currently participating in a clinical trial for your COPD, not including the Salford Lung Study (SLS)?

☐ Yes ☐ No ☐ Don't know/No answer

L3 Have you participated in a clinical trial for your COPD since the Salford Lung Study?

☐ Yes ☐ No ☐ Don't know/No answer

Thank you for completing this questionnaire!
