# Supplementary material for: Extending the data collection from a clinical trial: The Extended Salford Lung Study research cohort
Source: NPJ Prim Care Respir Med. 2023 Jan 18;33:4. doi: 10.1038/s41533-022-00322-7 (PMC9845305; doi:10.1038/s41533-022-00322-7)
Supplement: Supplementary file 2 — Reporting Summary [file 41533_2022_322_MOESM2_ESM.pdf]

nature portfolio

Corresponding author(s): Mark Fry, BSc
GSK, 980 Great West Road, Brentford, Middlesex, TW8 9GS, United Kingdom
Email: mark.a.fry@gsk.com

Last updated by author(s): Sep 9, 2022

Reporting Summary

Nature Portfolio wishes to improve the reproducibility of the work that we publish. This form provides structure for consistency and transparency in reporting. For further information on Nature Portfolio policies, see our Editorial Policies and the Editorial Policy Checklist.

Statistics

For all statistical analyses, confirm that the following items are present in the figure legend, table legend, main text, or Methods section.

n/a	Confirmed	
☐	☒	The exact sample size (*n*) for each experimental group/condition, given as a discrete number and unit of measurement
☒	☐	A statement on whether measurements were taken from distinct samples or whether the same sample was measured repeatedly
☒	☐	The statistical test(s) used AND whether they are one- or two-sided<br>*Only common tests should be described solely by name; describe more complex techniques in the Methods section.*
☒	☐	A description of all covariates tested
☒	☐	A description of any assumptions or corrections, such as tests of normality and adjustment for multiple comparisons
☐	☒	A full description of the statistical parameters including central tendency (e.g. means) or other basic estimates (e.g. regression coefficient) AND variation (e.g. standard deviation) or associated estimates of uncertainty (e.g. confidence intervals)
☒	☐	For null hypothesis testing, the test statistic (e.g. *F*, *t*, *r*) with confidence intervals, effect sizes, degrees of freedom and *P* value noted<br>*Give P values as exact values whenever suitable.*
☒	☐	For Bayesian analysis, information on the choice of priors and Markov chain Monte Carlo settings
☒	☐	For hierarchical and complex designs, identification of the appropriate level for tests and full reporting of outcomes
☒	☐	Estimates of effect sizes (e.g. Cohen's *d*, Pearson's *r*), indicating how they were calculated

Our web collection on statistics for biologists contains articles on many of the points above.

Software and code

Policy information about availability of computer code

Data collection	Primary care electronic health record data are coded using Read, Systematised Nomenclature of Medicine (SNOMED) and local Egton Medical Information Systems (EMIS) codes.
Data analysis	Descriptive data are reported, post-hoc analysis was performed using chi-square testing using Microsoft Excel.

For manuscripts utilizing custom algorithms or software that are central to the research but not yet described in published literature, software must be made available to editors and reviewers. We strongly encourage code deposition in a community repository (e.g. GitHub). See the Nature Portfolio guidelines for submitting code & software for further information.

Data

Policy information about availability of data

All manuscripts must include a data availability statement. This statement should provide the following information, where applicable:

- Accession codes, unique identifiers, or web links for publicly available datasets
- A description of any restrictions on data availability
- For clinical datasets or third party data, please ensure that the statement adheres to our policy

Anonymized individual participant data and study documents can be requested for further research from www.clinicalstudydatarequest.com. The secondary care data associated with this study are under license from NHS Digital and cannot be released as such.

Human research participants

Policy information about studies involving human research participants and Sex and Gender in Research.

Reporting on sex and gender	Both sexes were included in the study (60.0% female); no sex-specific analyses were conducted.
Population characteristics	See above
Recruitment	Patient recruitment (conducted 2018–2019) was general practitioner (GP) led. Participants consented to share primary care EHR data held by GP sites, as well as secondary care EHR data obtained from the NHS Digital Hospital Episodes Statistics databases covering admitted patient care, outpatient visits and Accident and Emergency attendances.
Ethics oversight	Ethical approval was granted by the North West - Greater Manchester East Research Ethics Committee (REC number: 17/NW/0122)

Note that full information on the approval of the study protocol must also be provided in the manuscript.

Field-specific reporting

Please select the one below that is the best fit for your research. If you are not sure, read the appropriate sections before making your selection.

☐ Life sciences ☒ Behavioural & social sciences ☐ Ecological, evolutionary & environmental sciences

For a reference copy of the document with all sections, see nature.com/documents/nr-reporting-summary-flat.pdf

Behavioural & social sciences study design

All studies must disclose on these points even when the disclosure is negative.

Study description	The Ext-SLS is an extension study of the Salford Lung Studies (SLS) combining clinical trial data from asthma and COPD patients with electronic health record (EHR) data and detailed, self-reported, information. This manuscript reports a descriptive analysis of the Ext-SLS primary care electronic health record and questionnaire data was conducted using N and % for categorical data; mean and standard deviation (SD) or median and inter-quartile range for continuous data.
Research sample	Patients with asthma and COPD who previously participated in the Salford Lung Studies (Phase IIIb, pragmatic, randomized, controlled clinical trials).
Sampling strategy	Participants were eligible for inclusion in the Ext-SLS if they were randomized to treatment in the original SLS and were able to provide written consent for data collection in the Ext-SLS. Patient recruitment (conducted 2018–2019) was general practitioner (GP) led. No sample size calculations were performed. This is an extension of a previously conducted clinical trial. Sample size calculations were conducted for the original trial, here we recruited as many participants from that trial as possible
Data collection	Patient recruitment (conducted 2018–2019) was general practitioner (GP) led. Participants consented to share primary care electronic health record (EHR) data held by GP sites, as well as secondary care EHR data obtained from the NHS Digital Hospital Episodes Statistics databases covering admitted patient care, outpatient visits and Accident and Emergency attendances. Participants completed disease-specific questionnaire booklets that captured information about their disease histories and management at the point of Ext-SLS consent.
Timing	Patients were recruited 2018–2019. Retrospective electronic health record data were collected to the earliest available record and prospective data are being collected periodically for up to 10 years from date of consent.
Data exclusions	No data were excluded from analysis.
Non-participation	1183 of a potential 7032 participants consented to take part. Overall 1147 participants completed questionnaires and had primary care data available.
Randomization	Randomization was performed in the original trial. The benefits of that randomization may have been lost in this extension, this is discussed in the manuscript.

Reporting for specific materials, systems and methods

We require information from authors about some types of materials, experimental systems and methods used in many studies. Here, indicate whether each material, system or method listed is relevant to your study. If you are not sure if a list item applies to your research, read the appropriate section before selecting a response.

Materials & experimental systems

n/a	Involved in the study
☒	☐ Antibodies
☒	☐ Eukaryotic cell lines
☒	☐ Palaeontology and archaeology
☒	☐ Animals and other organisms
☐	☒ Clinical data
☒	☐ Dual use research of concern

Methods

n/a	Involved in the study
☒	☐ ChIP-seq
☒	☐ Flow cytometry
☒	☐ MRI-based neuroimaging

Clinical data

Policy information about clinical studies

All manuscripts should comply with the ICMJE guidelines for publication of clinical research and a completed CONSORT checklist must be included with all submissions.

Clinical trial registration: NCT03152669

Study protocol: Study documents can be requested from www.clinicalstudydatarequest.com

Data collection: Patient recruitment (conducted 2018–2019) was general practitioner (GP) led. Participants consented to share primary care electronic health record (EHR) data held by GP sites, as well as secondary care EHR data obtained from the NHS Digital Hospital Episodes Statistics databases covering admitted patient care, outpatient visits and Accident and Emergency attendances. Participants completed disease-specific questionnaire booklets that captured information about their disease histories and management at the point of Ext-SLS consent.

Outcomes: A descriptive analysis of the Ext-SLS primary care EHR and questionnaire data was conducted using N and % for categorical data; mean and standard deviation or median and inter-quartile range for continuous data. Secondary care data were not available at time of analysis.
